# Predictors of falls and hospital admissions in people with cognitive impairment in day-care: role of multimorbidity, polypharmacy, and potentially inappropriate medication

**DOI:** 10.1186/s12877-022-03346-3

**Published:** 2022-08-18

**Authors:** Jennifer Scheel, Katharina Luttenberger, Elmar Graessel, André Kratzer, Carolin Donath

**Affiliations:** Department of Psychiatry and Psychotherapy, Center for Health Services Research in Medicine, Universitätsklinikum Erlangen, Friedrich-Alexander-Universität Erlangen-Nürnberg (FAU), Schwabachanlage 6, 91054 Erlangen, Germany

**Keywords:** Dementia, MCI, Multimorbidity, Polypharmacy, Potentially inappropriate medication, PRISCUS list, Falls, Hospitalization

## Abstract

**Background:**

Multimorbidity, polypharmacy, and potentially inappropriate medication (PIM) pose challenges for the care of people with cognitive impairment. The aim of the present study is to explore whether multimorbidity, polypharmacy, and PIM predict falls and hospital admissions in a sample of people with cognitive impairment in day-care centers in Germany.

**Methods:**

We used data from the German day-care study (multicenter longitudinal study, *n* = 433). Multimorbidity was defined as ≥ 2 chronic diseases. Polypharmacy was defined as prescriptions to ≥ 5 drugs. Potentially inappropriate medication was defined as scoring on the PRISCUS list. Binary logistic regression analyses were computed to determine whether multimorbidity, polypharmacy, and potentially inappropriate medication at t0 predicted falls and hospital admissions as outcomes at t1 (six months later).

**Results:**

The rate of multimorbidity and polypharmacy was 87.8% and 60.3%, respectively. 15.9% of the people with cognitive impairment received PIM / PRISCUS-listed drugs, 43.6% ACB-listed drugs, and 52.7% CNS depressant drugs. Falls and hospital admissions during follow-up were prevalent in 19.4% and 24.7% of the people with cognitive impairment. Both were significantly predicted by the total number of drugs (falls: OR = 1.152, *p* = 0.001, overall model: *p* < 0.001; hospital admissions: OR = 1.103, *p* = 0.020, overall model: *p* = 0.001), even if regression analyses were controlled for the number of comorbidities.

**Conclusions:**

Polypharmacy and potentially inappropriate medication are highly prevalent in people with cognitive impairment in German day-care centers. The number of drugs and appropriateness of medication seem to be crucial for the risk of falls and hospital admissions. Polypharmacy and PIM should be critically reviewed by healthcare providers and avoided as much as and whenever possible.

**Trial registration:**

ISRCTN16412551, 30 July 2014, registered partly retrospectively.

**Supplementary Information:**

The online version contains supplementary material available at 10.1186/s12877-022-03346-3.

## Background

Multimorbidity is often defined as ≥ 2 chronic diseases [1, 2]. Multimorbidity increases with age and is a common condition in older people [3, 4], particularly in older people with cognitive impairment (mild cognitive impairment and dementia) [3]. Multimorbidity is associated with poor quality of life [4], poor functional status [4], falls [5], hospitalization [3, 6, 7], and mortality [4].

Furthermore, multimorbidity is often associated with polypharmacy [7], which is common among older people [8]. There are multiple definitions of polypharmacy, most are numeric, and the most common in the community setting is ≥ 5 drugs per day [9]. Polypharmacy is related to various outcomes, such as drug interactions, adverse drug events, worsening of physical functioning/disability, falls [10–12], hospitalization [6, 11, 13], frailty [12, 13], and mortality [11, 12]. Yet, it has to be noted that a meta-review reported conflicting evidence for adverse drug events, drug interactions, falls, worsening of physical functioning/disability, and mortality [13].

Polypharmacy/higher number of drugs [13–15] and higher comorbidity [15, 16] are also associated with potentially inappropriate medication (PIM). PIM can be defined as “medications, which lack an indication, do not have sufficiently proven therapeutic effects or have an unfavorable ratio of risk of harm and intended benefit, and/or could be substituted by a safer drug” [17], this definition could also be transferred to potentially inappropriate polypharmacy. Appropriate polypharmacy can be defined as prescribing which reflects patients` clinical needs and is evidence based [18].

PIM are common in patients > 65 years (26–85%) [15, 19, 20] and also in people with cognitive impairment (10–60%) [16, 21]. PIM are related to a lower quality of life [20], low functional status [20], adverse drug events [22], and a higher risk of falls [16], hospital admission [15, 16, 22–24], and mortality [25].

Multimorbidity, polypharmacy, and PIM are associated with both falls and hospitalizations [3, 5, 6, 10–13, 15, 16, 22–24]. Falls are problematic for people with cognitive impairment, as they are associated with hospitalization [26], injury, loss of independence, and mortality [27]. The association between falls and hospitalizations is even stronger for people with cognitive impairment than for persons without dementia [28]. Hospitalizations are problematic for people with cognitive impairment, as they are associated with the deterioration of physical and mental abilities, negative psychological reactions, freedom-depriving measures, treatment-related complications, nosocomial infections, side effects of (new or additional) medications, delirium, falls, and unmet needs, such as unrecognized and inadequately treated pain. Furthermore, people with cognitive impairment are at risk of above-average lengths of hospital stays, conversion to a nursing home, and mortality [6, 29–34].

The association between polypharmacy and falls as well as polypharmacy and hospital admissions could be due to higher multimorbidity or higher use of PIM (e.g. medications with a high fall risk) in people with polypharmacy. Parsons et al. [14] stated in their review that inappropriate medication use in people with cognitive impairment is understudied at present. Most of the studies concerning multimorbidity, polypharmacy, and PIM in people with cognitive impairment have focused on people in nursing homes or hospitals. For people with cognitive impairment in day-care centers, data on these variables and their associations with falls and hospital admissions are still limited.

The aim of the present study is therefore to explore whether multimorbidity, polypharmacy, and PIM are predictors of falls and hospital admissions in a sample of people with cognitive impairment in day-care centers in Germany.

Specific aims:

1. Can the falls of people with cognitive impairment who use day-care centers be predicted by multimorbidity, polypharmacy, and PIM?
2. Can the hospital admissions of people with cognitive impairment who use day-care centers be predicted by multimorbidity, polypharmacy, and PIM?

## Methods

### Design, data collection, and sample

#### Design

The present study is a secondary analysis of the intention to treat sample (*n* = 433) from the German Day-care Study (DeTaMAKS, cluster-randomized, controlled, multicenter, prospective longitudinal study with a waitlist control group design, trial registration: ISRCTN16412551). For details of the study design see Behrndt et al. [35]. The study was carried out in 32 day-care centers in the following federal states of Germany: Baden-Wuerttemberg (Baden-Württemberg), Bavaria (Bayern), North Rhine-Westphalia (Nordrhein-Westfalen), Rhineland-Palatinate (Rheinland-Pfalz), Saxony-Anhalt (Sachsen-Anhalt), and Schleswig–Holstein (Schleswig–Holstein. The intervention group was offered MAKS therapy, which is a multimodal non-pharmacological training for people with cognitive impairment. In the control group, participants received “care as usual”. The original endpoints of the DeTaMAKS study were (1) cognition and activities of daily living of people with dementia and (2) subjective burden and well-being of informal caregivers [35].

#### Data collection

Data were obtained before intervention (MAKS therapy in the intervention group and “care as usual” / waiting period in the control group; t0, October 2014 to March 2015) and after 6 months of intervention (t1, March 2015 to October 2015). At t0, predictor variables (diagnoses and medications) were assessed from the day-care centers’ patients’ charts (all diagnoses and medication prescriptions which the caregivers reported to the day-care center). Furthermore, cognitive impairment variables were assessed by trained staff members working at the day-care centers. At t1, outcome variables (falls and hospital admissions) were assessed from the primary caregiver via telephone interviews and amended by information from the day-care centers’ patients’ charts.

#### Sample

Participants of this study were defined as people with cognitive impairment according to psychometric testing with the German versions of the Mini-Mental State Examination (MMSE) [36, 37] and the Montreal Cognitive Assessment (MoCA) [38]. First, the MMSE was conducted, and a score ≤ 23 was interpreted as dementia [39, 40]. If the MMSE score was ≥ 24, the MoCA was additionally conducted because the MMSE has a low sensitivity to detecting mild cognitive impairment (MCI) [36, 41–43]. A MoCA score of ≤ 22 was interpreted as MCI [35]. Inclusion criteria comprised use of day-care, cognitive impairment (mild cognitive impairment or dementia), having an informal caregiver, and informed consent. Exclusion criteria comprised complete blindness or deafness, severe dementia, cognitive decline due to diseases other than dementia (e.g. schizophrenia or Korsakov).

#### Dropouts

Until t1 (6 months after t0), 19 participants died, 35 moved to a nursing home, and 17 resigned from the study’s day-care centers. In the case of a dropout due to resign from the day-care center or conversion to a nursing home, data were still collected from the caregivers at t1 via telephone interview and referred to the whole 6-month period between t0 and t1. Supplementary data on the outcomes falls and hospital admissions from the day-care centers’ patients’ charts were collected until dropout for all three groups of dropout.

### Instruments

#### Assessment of cognitive impairment

##### Mini-mental state examination (MMSE)

The MMSE [36, 37] measures five areas of cognitive functioning: orientation, registration, attention/calculation, recall, and language. The score ranges from 0 to 30 points, with higher scores representing better cognitive performance. A MMSE score ≤ 23 indicates dementia [39, 40].

##### Montreal cognitive assessment (MoCA)

The MoCA [38] is a widely used, validated, and reliable screening tool for detecting MCI [41, 44]. The score ranges from 0 to 30 points, with higher scores representing better cognitive performance. The MoCA was only administered to participants with an MMSE score ≥ 24. A MoCA of ≤ 22 indicated dementia in the German Day-care Study [35].

##### Dementia diagnosis

The ICD-based diagnosis of dementia was assessed from the patients’ charts (F00 – F03) and included in the statistical analyses as a dichotomized variable.

#### Assessment of predictor variables (t0)

##### Comorbidities and multimorbidity

Multimorbidity was assessed with the number of comorbidities, the updated Charlson Comorbidity Index [45, 46], and the Functional Comorbidity Index (FCI) [47]:

##### Number of comorbidities

The comorbidities that existed in addition to psychometrically measured cognitive impairment were added up.

##### Updated Charlson Comorbidity Index

The original Charlson Comorbidity Index [45] was updated by Quan et al. [46] and is used to calculate the effect of any previous medical diagnoses on the mortality rate. Thus, it assigns weights to comorbidities according to their severity (with a range of 0–24 points).

##### Functional comorbidity index (FCI)

The FCI score [47] is a comorbidity index used to measure the influence of comorbidity on physical function. The FCI score is computed as the sum of 18 physical diagnoses (0–18 points).

##### History of diseases with a high risk of falls

Every participant was dichotomously rated on the presence or absence of a history of diseases with a high fall risk (Parkinson`s disease (ICD codes G20, G21, G23), multiple sclerosis (ICD code G35), stroke (ICD codes I60-64)).

##### Care level

In Germany, the care level depends on the need for care due to physical or mental disabilities and also determines which financial services the long-term care insurance will pay for. The classifications range from none (fully independent) to level 5 (most severe impairment to independence with special demands placed on nursing care).

##### Multimorbidity definition

According to Johnston et al. [1] and the NICE (National Institute for Health and Care Excellence) guidelines [2], multimorbidity is defined as ≥ 2 chronic diseases.

#### Medication, polypharmacy, and potentially inappropriate medication (PIM)

##### Total number of drugs, number of anti-dementia drugs, and number of non-anti-dementia psychiatric drugs

Anti-dementia drugs comprised memantine, acetylcholinesterase (ACH) inhibitors, and ginkgo biloba in this study. Non-anti-dementia psychiatric drugs comprised the ATC codes N05 and N06A, N06B, and N06C (e.g. antidepressants, antipsychotics, anxiolytics and sedative drugs). In addition to the number of drugs, dichotomous scores (yes/no) for all medication groups were built.

##### Polypharmacy definition

According to Masnoon et al. [9], receiving ≥ 5 drugs (regular prescribed medication) was defined as polypharmacy. Emergency medication, “as needed” (pro re nata / PRN) medication, over the counter medication, and topical / transdermal medication were not collected and were therefore not included in this definition.

##### Central nervous system (CNS) depressant drugs

All drugs were independently rated by two experts for clinical pharmacology on a scale with the following anchors: “very CNS depressant” (-2), “CNS depressant” (-1), “neutral” (0), “CNS activating” (+ 1), and “very CNS activating” (+ 2). The scores from each participant`s drugs were summed and formed the participant’s CNS depressant score. This approach was published in Lippert et al. [48], who also defined a CNS depressant score ranging from -1 to -2 as moderately depressant and a score ranging from -3 to -6 as strongly depressant. The list published by Lippert et al. [48] was updated in 2018. In addition to the CNS depressant score, the total number of CNS depressants drugs and a dichotomous score (yes/no) for CNS depressant drugs were assessed.

##### Drugs with anticholinergic cognitive burden (ACB)

The Anticholinergic Cognitive Burden (ACB) scale [49, 50] is a list introduced to measure anticholinergic burden in (geriatric) patients. Drugs are rated according to their anticholinergic effects as 1 (mild), 2 (moderate), or 3 (severe). The ACB scale was modified by adding definite anticholinergic drugs (biperiden, metixen, and maprotilin) with a score of 3 and by omitting trospium [51]. A total ACB sum score of ≥ 3 is considered clinically relevant [49]. In addition to the ACB score, the total number of drugs with anticholinergic cognitive burden**,** a dichotomous score of drugs with ACB (yes/no), and a dichotomous score created from the total ACB score of ≥ 3 (yes/no) were used.

##### PRISCUS list drugs

The PRISCUS (Latin for “old and venerable”) list [52] is a list of 83 drugs that are classified as potentially inappropriate drugs for people ≥ 65 years due to an increased risk of adverse drug events. The sum score [48] and a dichotomous score for PRISCUS list drugs (one or more PRISCUS list drugs prescribed, yes/no) was built. 14 persons whose ages were < 65 years were coded zero on all PRISCUS variables as the PRISCUS list was not applicable to them due to their age.

##### PIM definition

PIM was defined as receiving at least one drug from the PRISCUS list [52].

#### Assessment of the outcome variables (t1)

##### Falls

Falls were used as a dichotomized outcome variable (0 = no falls, 1 = at least one fall) for logistic regression analyses.

##### Hospital admissions

Hospital admissions were used as a dichotomized outcome variable (0 = no hospital admission, 1 = at least one hospital admission) for logistic regression analyses.

### Statistical analyses

Descriptive data (mean, standard deviation, range, median, mode, frequency) are provided for all variables. To explore whether multimorbidity, polypharmacy, and PIM are predictors of falls and hospital admissions, the statistical analyses comprised 3 steps: First, bivariate group comparisons (T-Tests and Chi^2^-tests) were computed. Second, all variables that differed in the pre-analysis between participants with vs. without an outcome event were tested for multicollinearity, as they are potential predictors in the regression analyses. As multicollinearity between predictor variables is problematic [53, 54], we chose only predictors that were correlated < 0.7 and additionally checked whether the variance inflation factors (VIFs) were < 5 [55]. Third, these predictors were included in binary logistic regression analyses for the two outcomes falls and hospital admissions. Fourth, we tested for whether the predictors were still significant after controlling the following variables for falls: age, sex, intervention group/control group, cognitive status, and history of diseases with a high risk of falls; and for hospital admissions: age, sex, intervention group/control group, cognitive status, falls. Statistical analyses were computed with the statistical analysis program SPSS 21. Findings were considered statistically significant at *p* < *0.05*.

## Results

### Description of socio-demographic data, dementia symptoms, comorbidities, and medication at t0

#### Socio-demographic data

A total of 433 people with cognitive impairment were included in the analyses. For socio-demographic data see Table 1.

**Table 1:** Socio-demographic data at t0 (*N* = 433)

Age	Mean ± SD (range)	81.6 ± 7.7 (43–99)
Sex	Female	62.1% (*n* = 269)
	Male	37.9% (*n* = 164)
Education	No school-leaving qualification	5.8% (*n* = 25)
	8–9 years of school education	71.8% (*n* = 311)
	10 years of school education	12.0% (*n* = 52)
	12–13 years of school education	4.8% (*n* = 21)
	University / university of applied science / college degree	5.5% (*n* = 24)

#### Cognitive symptoms

According to the MMSE and MoCA, 91 persons with MCI and 342 persons with dementia were included. 65.1% of the people with cognitive impairment were diagnosed with dementia (see Table 2 for details).

**Table 2:** Descriptions of cognitive symptoms, medications, and comorbidities at t0 (*N* = 433)

**Cognitive impairment, abilities, and symptoms**				
MMSE sum score at t0			Mean ± SD (range)	19.5 ± 4.7 (10–30)
MMSE dementia severity at t0			Score of 10–17 (moderate)	37.6% (*n* = 163)
			Score of 18–23 (mild)	41.3% (*n* = 179)
			Score of 24–30 (MCI)	21.1% (*n* = 91)
MoCA sum score (*n* = 91)			Mean ± SD (range)	18.0 ± 2.6 (10–22)
Dementia diagnosis			yes	65.1% (*n* = 282)
**Comorbidities and multimorbidity**				
Number of comorbidities in addition to dementia or MCI			Mean ± SD (range; modus)	2.5 ± 1.7 (0–8; 2)
Updated Charlson Comorbidity Index			Mean ± SD (range; modus)	2.3 ± 1.6 (0–8; 2)
FCI score			Mean ± SD (range)	1.7 ± 1.4 (0–6)
History of diseases with high risk of falls			yes	13.6% (*n* = 59)
Care level at t0			Care level 1 (%)	4.8%
			Care level 2 (%)	22.6%
			Care level 3 (%)	49.0%
			Care level 4 (%)	22.6%
			Care level 5 (%)	0.9%
**Medication**				
Total number of drugs			Mean ± SD (range)	5.2 ± 3.1 (0–15)
Psychiatric drugs	Anti-dementia drugs	Number	Mean ± SD (range)	0.3 ± 0.5 (0–2)
		Dichotomous (yes/no)	yes	30.9%
		Memantine (yes/no)	yes	13.2%
		ACH inhibitor (yes/no)	yes	17.1%
		Ginkgo biloba (yes/no)	yes	2.5%
	Non-anti-dementia drugs	Number	Mean ± SD (range)	0.7 ± 0.9 (0–4)
CNS depressant drugs		Dichotomous (yes/no)	yes	52.7%
		Number	Mean ± SD (range)	0.8 ± 1.0 (0–6)
		CNS depressant score	Mean ± SD (range)	-1.2 ± 1.6 (-10–1)
Drugs with anticholinergic cognitive burden (ACB)		Dichotomous (yes/no)	yes	43.6% (yes)
		Number	Mean ± SD (range)	0.6 ± 0.8 (0–5)
		ACB score	Mean ± SD (range)	0.9 ± 1.4 (0–9)
		ACB score of ≥ 3 (yes/no)	yes	16.2%
PRISCUS list drugs/PIM		Dichotomous (yes/no)	yes	15.9%
		Sum score (number)	Mean ± SD (range)	0.2 ± 0.4 (0–2)

Abbreviations: *ACB* Anticholinergic Cognitive Burden, *ACH* acetylcholinesterase, *FCI* Functional Comorbidity Index, *MMSE* Mini-Mental State Examination, *MoCA* Montreal Cognitive Assessment, *PIM* potentially inappropriate medication

#### Comorbidities

87.8% (*n* = 380) of the people with cognitive impairment had between 1 and 8 comorbidities with psychometrically measured dementia or MCI, so these people with cognitive impairment were included in the often used multimorbidity definition of ≥ 2 diseases [1] (see Table 2 for details).

The most frequent comorbidities were cardiovascular diseases (65.1%), endocrine, nutritional, and metabolic diseases (38.8%), diseases of the musculoskeletal system and connective tissue (27.5%), diseases of the nervous system (24.9%), diseases of the urogenital system (18.2%), and mental and behavioral disorders (14.8%).

#### Medication

**Fig. 1:**
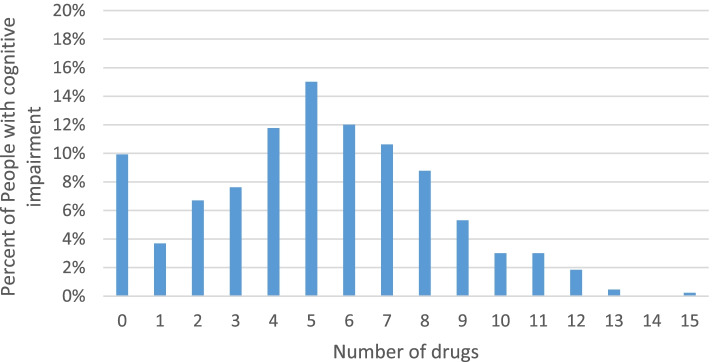
Total number of drugs

90.1% (*n* = 390) of the people with cognitive impairment were taking between 1 and 15 drugs (see Fig. 1 for details). 44.1% of the people with cognitive impairment were taking between 1 and 4 psychiatric drugs (exclusively anti-dementia drugs). 30.9% (*n* = 134) of the people with cognitive impairment were taking 1 or 2 anti-dementia drugs (see Table 2 for details).

##### Polypharmacy

60.3% (*n* = 271) of the people with cognitive impairment were taking ≥ 5 drugs and therefore fulfilled the polypharmacy definition according to Masnoon et al. [9].

##### CNS depressant drugs

52.7% (*n* = 228) of the people with cognitive impairment were taking CNS depressant drugs. The majority of the people with cognitive impairment with CNS depressant drugs were taking 1 CNS depressant drug (31.9%; *n* = 138). 52.0% of the people with cognitive impairment (*n* = 225) had a negative CNS depressant score, ranging from -1 to -10 (-1 to -2 = moderately depressant, -3 to -6 = strongly depressant [48]), 47.1% (*n* = 204) had a CNS depressant score of zero (“neutral”), and 0.9% (*n* = 4) had a positive CNS depressant score (CNS activating).

##### ACB score

43.6% (*n* = 189) of the people with cognitive impairment were taking between 1 and 5 drugs listed on the ACB scale. The ACB scores for this subsample ranged from 1 to 9. 16.2% (*n* = 70) of the people with cognitive impairment had a total ACB score of ≥ 3, which is considered to be clinically relevant [49].

##### PRISCUS list drugs/PIM

15.9% of the people with cognitive impairment were taking between 1 (13.8%) and 2 (2.1%) drugs listed on the PRISCUS list and therefore fulfilled our PIM definition (see Table 2 for details).

### Descriptions of outcome variables falls and hospital admissions at t1

#### Falls

84 people with cognitive impairment (19.4% of the whole sample) had experienced at least one fall. Of these 84 people with cognitive impairment, 53 (63.1% of falls) did not need medical care, 6 received ambulatory care (7.1% of falls) and 25 were admitted to the hospital (29.8% of falls). Thus, in the 31 cases that needed medical care due to a fall, 19% were ambulatory and 81% were stationary.

#### Hospital admissions

107 people with cognitive impairment (24.7%) had at least one hospital admission (79 people with cognitive impairment with 1 hospital admissions, 22 with 2, 3 with 3, and 3 with 4). Of these 144 hospital admissions, 25 (17.4%) were due to a fall, and 119 (82.6%) were due to reasons other than a fall (e.g. infections, cardiovascular diseases, or surgery). The total number of days in hospital was 7.2 ± 6.9 days with a range from 1 to 35 days and a median of 5 days.

### Specific aim 1: Can the falls of people with cognitive impairment who use day-care centers be predicted by multimorbidity, polypharmacy, and PIM?

#### Preanalysis

##### Bivariate analyses of group differences between people with cognitive impairment with and without a fall

There were group differences in the total number of drugs (t(431) = 3.805, *p* < 0.001), polypharmacy (Chi^2^ (1) = 9.435, *p* = 0.003), and anti-dementia drugs measured dichotomously (Chi^2^ (1) = 5.604, *p* = 0.019). Higher numbers of drugs were associated with falls. For details, see the additional files, Table S1.

##### Assessment of multicollinearity of potential predictor variables

A correlation of > 0.7 was found between the total number of drugs and polypharmacy (r = 0.795, *p* < 0.001). As high intercorrelations between predictor variables are problematic [53, 54], we decided to use the total number of drugs because this variable had the higher correlation with the outcome falls.

#### Main analysis: Binary regression analyses

After conducting the preanalysis and checking for multicollinearity (VIF = 1.029), two variables remained for the main analysis and were used as predictor variables for the outcome falls (no falls = 0; at least 1 fall = 1): the total number of drugs and anti-dementia drugs measured dichotomously (no antidementia drugs = 0; antidementia drugs = 1).

In the logistic regression analysis, the overall model was significant (Chi^2^ (2) = 17.388, *p* < 0.001, Nagelkerke`s R^2^ = 0.063). Only the predictor “total number of drugs” was significant (*p* = 0.001, OR = 1.152). The risk of falls was higher in people with cognitive impairment with a higher total number of drugs and increased by 15% for every additional drug. For details, see Table 3.

**Table 3:** Logistic regression model for the prediction of falls at t1

Predictor variable	Regression coefficient B (standard error)	Wald	Odds ratio	95% confidence interval	p single predictors
Total number of drugs	0.142 (0.042)	11.662	1.152	1.062–1.250	0.001
Anti-dementia drugs (dichotomous)	0.467 (0.256)	3.331	1.596	0.966–2.636	0.068

#### Sensitivity analyses

When the control variables age, sex, intervention/control group, cognitive status (MMST value at t0, dementia diagnosis at t0), number of comorbidities, and history of diseases with a high risk of falls were additionally added (1.030 ≤ VIF ≤ 1.405), the total number of drugs was still significant (*p* < 0.001, odds ratio = 1.173), and anti-dementia drugs measured dichotomously was still nonsignificant. Of the control variables, only “age” was significant (*p* = 0.027; people with cognitive impairment who had a fall were slightly older than those without a fall), and so was the overall model (Chi^2^ (9) = 26.754, *p* = 0.002, Nagelkerke`s R^2^ = 0.096).

When MCI status at t0 instead of the MMST value at t0 was used (1.032 ≤ VIF ≤ 1.404), the total number of drugs was still significant (*p* < 0.001, odds ratio = 1.177), and anti-dementia drugs measured dichotomously was still nonsignificant. Of the control variables, only “age” was significant (*p* = 0.024; people with cognitive impairment who had a fall were slightly older than people with cognitive impairment without a fall), and so was the overall model (Chi^2^ (9) = 26.396, *p* = 0.002, Nagelkerke`s R^2^ = 0.094).

When performing the regression analyses excluding deceased participants, there were no differences regarding predictors and the final models (except marginal changes in the decimal places).

When performing the regression analyses with only the dementia subgroup, there were no differences regarding predictors and the final models (except marginal changes in the decimal places).

### Specific aim 2: Can the hospital admissions of people with cognitive impairment who use day-care centers be predicted by multimorbidity, polypharmacy, and PIM?

#### Preanalysis

Bivariate analyses of group differences between people with cognitive impairment with and without a hospital admission.

There were six group differences between participants with and without a hospital admission, namely, the total number of drugs (t(431) = 3.596, *p* < 0.001), polypharmacy (Chi^2^ (1) = 9.453, *p* = 0.002), the number of CNS depressant drugs (t(431) = 3.091, *p* = 0.002), the CNS depressant score (t(431) = -2,778, *p* = 0.006), the dichotomous CNS depressant score (Chi^2^ (1) = 6.768, *p* = 0.010), and the dichotomous score created from the total ACB score of ≥ 3 (Chi^2^ (1) = 6.936, *p* = 0.010). Generally higher numbers of drugs and higher CNS depressant scores were associated with hospital admissions. For details, see the additional files, Table S2.

Assessment of multicollinearity of the potential predictor variables.

A correlation of > 0.7 was found for a) the total number of drugs and polypharmacy (r = 0.795, *p* < 0.001) as well as for b) the number of CNS depressant drugs, the CNS depressant score, and the dichotomous CNS depressant score (0.725 ≤ r ≤ 952, *p* < 0.001). We chose a) the total number of drugs and b) the number of CNS depressant drugs for the multivariate analysis because these variables had the strongest correlations with the outcome hospital admissions.

#### Main analysis: Binary regression analyses

After we computed the preanalysis and checked for multicollinearity (1.309 ≤ VIF ≤ 1.587), three predictor variables remained for the main analysis and were used as predictors of the outcome hospital admissions (no hospital admissions = 0; at least 1 hospital admission = 1): the total number of drugs, the number of CNS depressant drugs, and the dichotomous score created from the total ACB score of ≥ 3.

In the logistic regression analysis, the overall model was significant (Chi^2^ (3) = 15.702, *p* = 0.001, Nagelkerke`s R^2^ = 0.053). The total number of drugs (*p* = 0.020, OR = 1.103) was significantly associated with the outcome hospital admissions, whereas the number of CNS depressant drugs (*p* = 0.360, OR = 1.130) and the dichotomous score created from the total ACB score of ≥ 3 (*p* = 0.347, OR = 1.363) were not. The risk of hospital admissions was higher in people with cognitive impairment with a higher total number of drugs – the risk of hospital admission increased by 10% for every additional drug. For details, see Table 4.

**Table 4:** Logistic regression model for predicting hospital admissions at t1

Predictor variable	Regression coefficient B (standard error)	Wald	Odds ratio	95% confidence interval	p
Total number of drugs	0.098 (0.042)	5.423	1.103	1.016–1.199	0.020
Number of CNS depressant drugs	0.122 (0.134)	0.839	1.130	0.870–1.469	0.360
Dichotomous score created from the total ACB score of ≥ 3 (yes/no)	0.309 (0.329)	0.885	1.363	0.715–2.596	0.347

*Abbreviations:* *ACB* Anticholinergic Cognitive Burden, *CNS* Central nervous system

#### Sensitivity analyses

When the control variables age, sex, intervention/control group, cognitive status (MMST value at t0, dementia diagnosis at t0), and number of comorbidities were added to the model (1.021 ≤ VIF ≤ 1.651), none of these variables were significant, but the total number of drugs remained a significant predictor (*p* = 0.036, odds ratio = 1.101).

When the variable “falls” was added as another control variable (1.021 ≤ VIF ≤ 1.652), the total number of drugs was no longer a significant predictor (neither were the number of CNS depressant drugs or the dichotomous score created from the total ACB score of ≥ 3). In this case, falls was a significant predictor (*p* < 0.001), and the overall model was also significant (Chi^2^ (10) = 39.895, *p* < 0.001, Nagelkerke`s R^2^ = 0.131). Experiencing a fall increased the risk of a hospital admission more than threefold (odds ratio of 3.467).

When MCI status at t0 instead of the MMST value at t0 was used (1.022 ≤ VIF ≤ 1.661), none of the control variables were significant, but the total number of drugs remained a significant predictor (*p* = 0.041, odds ratio = 1.099). When the variable “falls” was added as another control variable (1.022 ≤ VIF ≤ 1.662), the total number of drugs was no longer a significant predictor (neither were the number of CNS depressant drugs or the dichotomous score created from the total ACB score of ≥ 3). In this case, falls was a significant predictor (*p* < 0.001), and the overall model was also significant (Chi^2^ (10) = 38.765, *p* < 0.001, Nagelkerke`s R^2^ = 0.127). Experiencing a fall increased the risk of a hospital admission more than threefold (odds ratio of 3.413).

When performing the regression analyses excluding deceased participants, there were no differences regarding predictors and the final models (except marginal changes in the decimal places).

When performing the regression analyses with only the dementia subgroup, the final results remained the same. The only difference was that instead of the number of the CNS depressant drugs, the CNS depressant score was used.

## Discussion

Disease load and medication use was high, whereas the numbers of participants with falls or hospital admissions were rather low. Medication seemed to be crucial for both outcome variables, falls and hospital admissions, as higher number of drugs were associated with a higher risk of falls and hospital admissions.

This is similar to recent studies that found that falls were associated with polypharmacy [10, 11] and a larger number of medications [10, 12, 56] and that hospital admissions were associated with polypharmacy [13], number of medication [11], and ≥ 7 drugs [6]. As mentioned in the introduction, hospitalizations constitute a relevant risk for severe consequences for people with cognitive impairment. Furthermore, a hospital admission is again a risk for further inappropriate medication, and Perez et al. [15] found a 72% increase in the risk of PIM after a hospital admission compared with before. Therefore, critically reviewing a patient’s medications and polypharmacy seems to be very important for reducing hospital admissions and their potential negative consequences.

In the current study, we did not find that PIM was related to falls (as Renom-Guiteras et al. [16] found) or hospital admissions (as other studies [15, 16, 22–24] have found). We also did not find that any of the comorbidity/multimorbidity variables were related to falls (as Ek et al. [5] found) or to hospital admissions (as Shepherd et al. [6] found). That PIM and multimorbidity were not related to falls or hospital admissions in the present study could be due to 1) the small subsample sizes of people with cognitive impairment with falls (19.4%) and hospital admissions (24.7%), which can make it difficult for a single predictor to reach significance and might be due to the duration of observations in the present study, which might have been too short, or 2) the constitution of our sample of people with cognitive impairment, who showed quite good physical functioning, even in the face of multimorbidity. Good physical functioning is a protective factor against the risk of hospitalization [6] and might counterbalance multimorbidity in the present sample.

It has been well known for years that polypharmacy is harmful to people with cognitive impairment. The NICE guidelines [57] warn against problematic polypharmacy. Also, the WHO (World Health Organization) mentions polypharmacy in their third global patient safety challenge “Medication without harm” [58]. However, polypharmacy is an ongoing problem: The rate of polypharmacy (≥ 5 drugs) was 60.3% for the people with cognitive impairment in day-care centers in the present study. If the stricter WHO definition (≥ 4 drugs) [58] had been applied, the percentage would have been even higher. Polypharmacy (≥ 5 drugs) rates of older people were slightly lower in primary care and in the general population (27%-59% and 40%-67%, respectively) [8], slightly higher in nursing home residents (66.2%) [48] and the highest for hospital care (46%-84%) [8], especially in acute medical units (80%) [20]. In some cases, polypharmacy might just be a marker of the health status, multimorbidity, or frailty of a person with cognitive impairment. In other cases, it might be an independent risk factor and might indicate potentially inappropriate medication (PIM). The problem of bias by indication – falls and hospital admissions might not be associated only with polypharmacy but also with the health conditions that lead to medication prescriptions and polypharmacy – has to be considered. Yet in our study, neither falls nor hospital admissions were related to the multimorbidity or comorbidity variables and regression analyses were controlled for the number of comorbidities. Falls and hospital admissions were (still) related only to the medication variables.

It has also been well known for years that PIM and particular drug groups, such as drugs with anticholinergic cognitive burden and CNS depressant drugs, can be harmful to people with cognitive impairment. The German S3-Guideline [59] (“S3-Leitlinie,” equivalent to the medical guidelines from the NICE Institute [60]) discourages the use of anticholinergics and PRISCUS list drugs in people with cognitive impairment. However, 15.9% of the people with cognitive impairment in the present study received PRISCUS list drugs (i.e. PIM), 43.6% received ACB-listed drugs, and 52.7% received CNS depressant drugs. As medication use and PIM use are higher in nursing home residents than in community-dwelling persons [16, 21], the percentages in the present study were slightly lower than those found by Lippert et al. [48] for German nursing home residents (29.5%, 50.4%, and 61.9%, respectively). Yet, the mean CNS depressant score in our sample was -1.2 ± 1.6, which is comparable to Lippert et al. [48] (-1.4 ± 1.6) and is interpreted as a moderate CNS depressant [48]. Yet, we did not find the association between falls and CNS depressant drugs that Lippert et al. [48] found. In line with our results regarding the PRISCUS list, taking at least one PRISCUS list drug was reported for 19.8–22.1% of people with cognitive impairment living at home [61] and 25.9% of patients discharged from geriatric units (93.7% living at home) [19]. The proportion of participants receiving PIM in our study is also only marginally lower than measured by the STOPP criteria in the study of Ryan et al. [62], who reported 21.4% in a primary care sample. In line with our results for the ACB scale, Pfistermeier et al. [51] found that 46.3% of geriatric patients took at least one ACB-listed drug. Yet, we did not find that falls were related to anticholinergics as Perttila et al. [56] found.

In the present study, polypharmacy was found to be a risk factor for both falls and hospitalizations in people with cognitive impairment. In general, older people are more susceptible to harmful drug effects (e.g. adverse drug reactions) because of age-related changes and pathologies [63]. Furthermore, people with cognitive impairment might even be at a higher risk of polypharmacy and PIM, as dementia and particularly BPSD (behavioral and psychological symptoms of dementia) are often treated with (additional) medical drugs, such as antipsychotics (10% of the people with cognitive impairment in ambulatory dementia care [64] and more than 50% of the people with cognitive impairment in nursing homes [48, 65]), which might be PIM [17]. Additionally, their dementia symptoms might deter people with cognitive impairment from playing an active role in their medication regimen due to comprehension problems and communication problems.

Therefore, the optimization of drug prescriptions is essential. Kouladjian et al. [66] reported in their review that medication regimens of aged people / people with cognitive impairment should be carefully assessed by clinicians with special attention to inappropriate medication and polypharmacy [66]. Patterson et al. [67] concluded in their Cochrane Review that interventions to improve polypharmacy seem beneficial regarding inappropriate prescribing and medication-related problems. Harrison et al. [68] reported in their review that deprescribing psychotropic medications for BPSD and insomnia can be effective for people living in long-term aged care.

Furthermore, there are psychosocial intervention strategies that should be tried first or can help reduce already existing prescriptions in people with dementia [59].

Our study has several strengths. First, we were able to provide a detailed description of multimorbidity and polypharmacy in a large sample of people with cognitive impairment in day-care centers. The data were longitudinal and comprised validated instruments and validated definitions of multimorbidity and polypharmacy (comparability). Second, external validity was given, as day-care centers from all over Germany were included.

On the other hand, our study has some limitations. First, medication and diagnosis data were collected only at t0, and the medication dosage was not available. In future studies, medication data should be collected throughout the study so changes can be included. Second, the data were largely assessed via self-report by the caregivers: The diagnoses and medication prescriptions from the day-care centers` patient charts were reported to the day-care center by the caregivers (missings and vagueness in self-report possible). Third, the sizes of the subsamples with falls and hospital admissions were small, so it was difficult for single predictors to reach significance. Furthermore, falls were analysed as a dichotomous outcome and the data regarding falls of dropouts is stronger relying on the information of the caregivers than the data of the non-dropouts, as supplementary data on the outcomes falls and hospital admissions from the day-care centers’ patients’ charts could only be collected until dropout. Fourth, the study population comprised a wide range of cognitive impairment from MCI to moderate dementia. Furthermore, the findings of this study may not be generalizable to younger patients as the study population was quite old.

## Conclusions

Though the risks are known and reduction strategies exist, polypharmacy and PIM are still common in people with cognitive impairment. In the present study, polypharmacy and/or PIM were present in almost two thirds of the people with cognitive impairment. Polypharmacy and PIM are associated with various risks and negative consequences for people with cognitive impairment, such as falls and hospital admissions, which are again related to further negative consequences.

Practical implication: Due to their confirmed associations with falls and hospital admissions, polypharmacy and PIM should be critically reviewed by healthcare providers and avoided as much as and whenever possible. This can be accomplished by I) the deprescribing of drugs, II) the avoidance of PIM, and III) the application of non-pharmacological therapies that have no side effects. As polypharmacy and/or PIM was present in a large proportion of people with cognitive impairment, a large impact can be made in the reduction of falls and hospital admissions in people with cognitive impairment.

## Supplementary Information

**Additional file 1:** **Table S1.** Bivariateanalyses of group differences between PWCI with and without a fall. **Table S2.** Bivariateanalyses of group differences between PWCI with and without a hospitaladmission.

## Data Availability

The data set supporting the conclusions of this article is available from the corresponding author upon reasonable request.

## Abbreviations

ACB: Anticholinergic Cognitive Burden
ACH: Acetylcholinesterase inhibitor
BPSD: Behavioral and psychological symptoms of dementia
CNS: Central nervous system
FCI: Functional Comorbidity Index
MMSE: Mini-Mental State Examination
MoCA: Montreal Cognitive Assessment
NICE: National Institute for Health and Care Excellence
PIM: Potentially inappropriate medication
WHO: World Health Organization
