# Supplementary material for: Predictors of falls and hospital admissions in people with cognitive impairment in day-care: role of multimorbidity, polypharmacy, and potentially inappropriate medication
Source: BMC Geriatr. 2022 Aug 18;22:682. doi: 10.1186/s12877-022-03346-3 (PMC9387045; doi:10.1186/s12877-022-03346-3)
Supplement: Supplementary file 1 — Additional file 1: Table S1. Bivariateanalyses of group differences between PWCI with and without a fall. Table S2. Bivariateanalyses of group differences between PWCI with and without a hospitaladmission. [file 12877_2022_3346_MOESM1_ESM.docx]

Additional files

Table S1: Bivariate analyses of group differences between PWCI with and without a fall

|  | | | | | **PWCI with at least 1 fall (n=84)** | **PWCI without fall (n=349)** | **Test** |
| --- | --- | --- | --- | --- | --- | --- | --- |
| **Comorbidities and multimorbidity** | | | | | | | |
| Number of comorbidities in addition to dementia or MCI | | | | M±SD | 2.5±1.6 | 2.5 ±1.7 | t(431)=0.121, p=0.904 |
| Multimorbidity (≥2 chronic diseases) | | | | % yes | 90.5% (n=76) | 87.1% (n=304) | Chi²(1)=0.716, p=0.463 |
| Updated Charlson Comorbidity Index | | | | M±SD | 2.3±1.6 | 2.3±1.6 | t(431)=0.036, p=0.972 |
| FCI score | | | | M±SD | 1.8±1.2 | 1.7 ±1.4 | t(431)=1.037, p=0.301 |
| History of diseases with high risk of falls | | | | % yes | 17.9% (n=15) | 12.6% (n=44) | Chi²(1)=1.585, p=0.217 |
| Care level at t0 | | | Care level 1 | % yes | 3.6% (n=3) | 5.2% (n=18) | Chi²(4)=4.057, p=0.388 |
|  |  |  | Care level 2 | % yes | 23.8% (n=20) | 22.3% (n=78) |  |
|  |  |  | Care level 3 | % yes | 56.0% (n=47) | 47.3% (n=165) |  |
|  |  |  | Care level 4 | % yes | 16.7% (n=14) | 24.1% (n=84) |  |
|  |  |  | Care level 5 | % yes | 0% (n=0) | 1.1% (n=4) |  |
| **Medication** | | | | | | | |
| Total number of drugs | | | | M±SD | **6.4±3.0** | **4.9±3.0** | **t(431)=3.805, p<0.001** |
| Polypharmacy (≥5 drugs) | | | | % yes | **75.0% (n=63)** | **56.7% (n=198)** | **Chi²(1)=9.435, p=0.003** |
| Psychiatric drugs | Anti-dementia drugs | Number | | M±SD | 0.4 ±0.5 | 0.3 ±0.5 | t(431)=1.934, p=0.055 |
|  |  | Dichotomous (yes/no) | | % yes | **41.7% (n=35)** | **28.4% (n=99)** | **Chi²(1)=5.604, p=0.019** |
|  |  | Memantine (yes/no) | | % yes | 17.9% (n=15) | 12.0% (n=42) | Chi²(1)=2.008, p=0.207 |
|  |  | ACH inhibitor (yes/no) | | % yes | 20.2% (n=17) | 16.3% (n=57) | Chi²(1)=0.729, p=0.420 |
|  |  | Ginkgo biloba (yes/no) | | % yes | 4.8% (n=4) | 2.0% (n=7) | Chi²(1)=2.077, p=0.236 |
|  | Non-anti-dementia drugs | Number | | M±SD | 0.8 ±0.9 | 0.7±0.9 | t(431)=0.877, p=0.381 |
| CNS depressant drugs | | Dichotomous (yes/no) | | % yes | 58.3% (n=49) | 51.3% (n=179) | Chi²(1)=1.348, p=0.274 |
|  |  | Number | | M±SD | 0.9±1.0 | 0.8±1.0 | t(431)=1.037, p=0.300 |
|  |  | CNS depressant score | | M±SD | -1.3±1.5 | -1.1±1.6 | t(431)=-1.013, p=0.312 |
| Drugs with anticholinergic cognitive burden (ACB) | | ACB scale drugs (yes/no) | | % yes | 48.8% (n=41) | 42.4% (n=148) | Chi²(1)=1.128, p=0.327 |
|  |  | Number of ACB scale drugs | | M±SD | 0.7±0.9 | 0.6±0.8 | t(431)=1.249, p=0.212 |
|  |  | ACB score | | M±SD | 1.0±1.5 | 0.9±1.4 | t(431)=0.438, p=0.662 |
|  |  | ACB score of ≥3 (yes/no) | | % yes | 15.5% (n=13) | 16.3% (n=57) | Chi²(1)=0.037, p=0.872 |
| PRISCUS list drugs/PIM | | PRISCUS list drugs (yes/no) | | % yes | 19.0% (n=16) | 15.2% (n=53) | Chi²(1)=0.754, p=0.407 |
|  |  | Number of PRISCUS list drugs | | M±SD | 0.2±0.4 | 0.2±0.4 | t(431)=0.521, p=0.603 |

Table S2: Bivariate analyses of group differences between PWCI with and without a hospital admission

|  | | | | | **PWCI with at least 1 hospital admission (n=107)** | **PWCI without any hospital admissions (n=326)** | **Test** |
| --- | --- | --- | --- | --- | --- | --- | --- |
| **Comorbidities and multimorbidity** | | | | | | | |
| Number of comorbidities in addition to dementia or MCI | | | | M±SD | 2.7±1.7 | 2.4 ±1.7 | t(431)=1.278, p=0.202 |
| Multimorbidity (≥2 chronic diseases) | | | | % yes | 90.7% (n=97) | 86.8% (n=283) | Chi²(1)=1.108, p=0.315 |
| Updated Charlson Comorbidity Index | | | | M±SD | 2.3±1.6 | 2.2±1.6 | t(431)=0.180, p=0.857 |
| FCI score | | | | M±SD | 1.8±1.2 | 1.7 ±1.4 | t(431)=0.670, p=0.503 |
| History of diseases with high risk of falls | | | | % yes | 16.8% (n=18) | 12.6% (n=41) | Chi²(1)=1.234, p=0.329 |
| Care level at t0 | | | Care level 1 | % yes | 3.7% (n=4) | 5.2% (n=17) | Chi²(4)=5.678, p=0.223 |
|  |  |  | Care level 2 | % yes | 23.4% (n=25) | 22.4% (n=73) |  |
|  |  |  | Care level 3 | % yes | 42.1% (n=45) | 51.2% (n=167) |  |
|  |  |  | Care level 4 | % yes | 29.0% (n=31) | 20.6% (n=67) |  |
|  |  |  | Care level 5 | % yes | 1.9% (n=2) | 0.6% (n=2) |  |
| **Medication** | | | | | | | |
| Total number of drugs | | | | M±SD | **6.1±3.0** | **4.9±3.0** | **t(431)=3.596, p<0.001** |
| Polypharmacy (≥5 drugs) | | | | % yes | 72.9% (n=**78)** | 56.1% (n=**183)** | **Chi²(1)=9.453, p=0.002** |
| Psychiatric drugs | Anti-dementia drugs | Number | | M±SD | 0.3 ±0.4 | 0.4 ±0.5 | t(431)=-1.505, p=0.134 |
|  |  | Dichotomous (yes/no) | | % yes | 27.1% (n=29) | 32.2% (n=105) | Chi²(1)=0.983, p=0.338 |
|  |  | Memantine (yes/no) | | % yes | 15.0% (n=16) | 12.6% (n=41) | Chi²(1)=0.398, p=0.621 |
|  |  | ACH inhibitor (yes/no) | | % yes | 11.2% (n=12) | 19.0% (n=62) | Chi²(1)=3.462, p=0.075 |
|  |  | Ginkgo biloba (yes/no) | | % yes | 0.9% (n=1) | 3.1% (n=10) | Chi²(1)=1.480, p=0.307 |
|  | Non-anti-dementia drugs | Number | | M±SD | 0.8 ±1.0 | 0.6±0.9 | t(431)=1.735, p=0.084 |
| CNS depressant drugs | | Dichotomous (yes/no) | | % yes | **63.6% (n=68)** | **49.1% (n=160)** | **Chi²(1)=6.768, p=0.010** |
|  |  | Number | | M±SD | **1.1±1.1** | **0.7±0.9** | **t(431)=3.091, p=0.002** |
|  |  | CNS depressant score | | M±SD | **-1.6±1.7** | **-1.1±1.5** | **t(431)=-2.778, p=0.006** |
| Drugs with anticholinergic cognitive burden (ACB) | | ACB scale drugs (yes/no) | | % yes | 47.7% (n=51) | 42.3% (n=138) | Chi²(1)=0.931, p=0.369 |
|  |  | Number of ACB scale drugs | | M±SD | 0.7±0.9 | 0.6±0.8 | t(431)=1.062, p=0.289 |
|  |  | ACB score | | M±SD | 1.2±1.7 | 0.8±1.4 | t(431)=1.876, p=0.0.63 |
|  |  | ACB score of ≥3 (yes/no) | | % yes | **24.3% (n=26)** | **13.5% (n=44)** | **Chi²(1)=6.936, p=0.010** |
| PRISCUS list drugs/PIM | | PRISCUS list drugs (yes/no) | | % yes | 19.6% (n=21) | 14.7% (n=48) | Chi²(1)=1.445, p=0.286 |
|  |  | Number of PRISCUS list drugs | | M±SD | 0.2±0.5 | 0.2±0.4 | t(431)=1.318, p=0.189 |
